# Exposure-Response Estimates for Diesel Engine Exhaust and Lung Cancer Mortality Based on Data from Three Occupational Cohorts

**DOI:** 10.1289/ehp.1306880

**Published:** 2013-11-22

**Authors:** Roel Vermeulen, Debra T. Silverman, Eric Garshick, Jelle Vlaanderen, Lützen Portengen, Kyle Steenland

**Affiliations:** 1 Division of Environmental Epidemiology, Institute for Risk Assessment Sciences, Utrecht University, Utrecht, the Netherlands; 2 Occupational and Environmental Epidemiology Branch, Division of Cancer Epidemiology and Genetics, National Cancer Institute, National Institutes of Health, Department of Health and Human Services, Bethesda, Maryland, USA; 3 Pulmonary and Critical Care Medicine Section, Medical Service, Veterans Affairs Boston Healthcare System; Channing Division of Network Medicine, Brigham and Women’s Hospital and Harvard Medical School, Boston, Massachusetts, USA; 4 Section of Environment and Radiation, International Agency for Research on Cancer, Lyon, France; 5 Department of Environmental and Occupational Health, Rollins School of Public Health, Emory University, Atlanta, Georgia, USA

## Abstract

Background: Diesel engine exhaust (DEE) has recently been classified as a known human carcinogen.

Objective: We derived a meta-exposure–response curve (ERC) for DEE and lung cancer mortality and estimated lifetime excess risks (ELRs) of lung cancer mortality based on assumed occupational and environmental exposure scenarios.

Methods: We conducted a meta-regression of lung cancer mortality and cumulative exposure to elemental carbon (EC), a proxy measure of DEE, based on relative risk (RR) estimates reported by three large occupational cohort studies (including two studies of workers in the trucking industry and one study of miners). Based on the derived risk function, we calculated ELRs for several lifetime occupational and environmental exposure scenarios and also calculated the fractions of annual lung cancer deaths attributable to DEE.

Results: We estimated a lnRR of 0.00098 (95% CI: 0.00055, 0.0014) for lung cancer mortality with each 1-μg/m^3^-year increase in cumulative EC based on a linear meta-regression model. Corresponding lnRRs for the individual studies ranged from 0.00061 to 0.0012. Estimated numbers of excess lung cancer deaths through 80 years of age for lifetime occupational exposures of 1, 10, and 25 μg/m^3^ EC were 17, 200, and 689 per 10,000, respectively. For lifetime environmental exposure to 0.8 μg/m^3^ EC, we estimated 21 excess lung cancer deaths per 10,000. Based on broad assumptions regarding past occupational and environmental exposures, we estimated that approximately 6% of annual lung cancer deaths may be due to DEE exposure.

Conclusions: Combined data from three U.S. occupational cohort studies suggest that DEE at levels common in the workplace and in outdoor air appear to pose substantial excess lifetime risks of lung cancer, above the usually acceptable limits in the United States and Europe, which are generally set at 1/1,000 and 1/100,000 based on lifetime exposure for the occupational and general population, respectively.

Citation: Vermeulen R, Silverman DT, Garshick E, Vlaanderen J, Portengen L, Steenland K. 2014. Exposure-response estimates for diesel engine exhaust and lung cancer mortality based on data from three occupational cohorts. Environ Health Perspect 122:172–177; http://dx.doi.org/10.1289/ehp.1306880

## Introduction

Recently, a working group of the International Agency for Research on Cancer (IARC) Monograph Series reviewed the scientific evidence regarding the carcinogenicity of diesel engine exhaust (DEE). The Working Group concluded that DEE is a cause of lung cancer (Group 1: carcinogenic to humans) based on human, animal, and experimental evidence (Benbrahim-Tallaa et al. 2012). Given that large populations of workers are exposed to DEE in the workplace and that urban populations are exposed to low levels of DEE in the ambient environment, the potential public health impact of DEE exposure may be considerable. For example, Rushton et al. (2012) recently estimated that occupational DEE exposure in the United Kingdom was the third most important occupational contributor to the lung cancer burden after asbestos and silica exposure.

At the time of the IARC evaluation, three U.S. occupational cohort studies of cumulative exposure to elemental carbon (EC; a marker of DEE) and lung cancer mortality had reported exposure–response estimates, including a study of non-metal miners (198 lung cancer deaths) (Attfield et al. 2012; Silverman et al. 2012) and two independent studies of trucking industry workers (779 and 994 lung cancer deaths, respectively) (Garshick et al. 2012; Steenland et al. 1998). A fourth cohort study of potash miners (68 lung cancers) with EC exposure–response data was published after the IARC evaluation (Mohner et al. 2013). To clarify the public health impacts of DEE exposures, we conducted a formal meta-regression to derive an exposure–response estimate for cumulative EC and lung cancer mortality and used it to estimate excess lifetime lung cancer mortality for environmental and occupational exposures and attributable fractions of lung cancer deaths due to DEE.

## Methods

*Data*. We performed, as part of the IARC evaluation, a detailed literature search using MEDLINE (http://www.ncbi.nlm.nih.gov/pubmed/). Search terms included “diesel,” “elemental carbon,” and “lung cancer.” The reference lists of candidate studies and review articles were also manually examined to find any additional relevant studies. Studies were included in the meta-regression *a*) if DEE exposure was expressed as cumulative EC in the exposure–response analyses, *b*) if an appropriate unexposed/low exposed reference group was used, and *c*) if no major methodological shortcomings were noted. The great majority of studies did not include quantitative exposure–response data. There were only three studies identified that met our criteria (Garshick et al. 2012; Silverman et al. 2012; Steenland et al. 1998). One additional study, with quantitative exposure–response data was published after the IARC evaluation and initial literature search (Mohner et al. 2013).

We excluded the study by Mohner et al. (2013) because the mean cumulative EC exposure in the reference exposure category (624 μg/m^3^-years) was higher than almost all of the nonreference exposure categories of the other studies, the cohort included only 68 lung cancer deaths, and the derivation of the EC exposure metric was not described in detail. In addition, we had concerns about the method used to adjust for previous employment in uranium mining because the results were dramatically different from an earlier analysis of the same data (Neumeyer-Gromen et al. 2009). However, we did include data from Mohner et al. (2013) in a sensitivity analysis of the obtained ERC (see Supplemental Material), with and without a correction of the reported relative risk (RR) estimates for the high level of exposure in the referent group in that study.

From the three studies included in the primary meta-regression, we extracted categorical RRs [hazard ratios (HRs), odds ratios (ORs)] from the main analyses presented by the authors of each study. From the nested case–control study by Steenland et al. (1998) of trucking industry workers, we used ORs for cumulative EC exposure categories with a 5-year lag. Steenland et al. (1998) included 994 lung cancer deaths and 1,085 controls. All cases and controls had died in 1982–1983 and were long-term Teamsters enrolled in the pension system. Cases and controls were divided by job categories based on the longest held job. In 1988–1989, submicrometer EC was measured in 242 samples covering the major job categories in the trucking industry (Steenland et al. 1998). Estimates of past exposure to EC, for participants in the epidemiologic study were made by assuming that *a*) average 1990 levels for a job category could be assigned to all subjects in that job category, and *b*) levels prior to 1990 were directly proportional to vehicle miles traveled by heavy duty trucks and the estimated emission levels of diesel engines.

From the cohort study of trucking industry workers by Garshick et al. (2012), we used HRs for cumulative EC exposure categories with a 5-year lag based on analyses that excluded mechanics. In that study, work records were available for 31,135 male workers employed in the unionized U.S. trucking industry in 1985. Mortality was ascertained through the year 2000 and included 779 lung cancer deaths. From 2001 through 2006 a detailed exposure assessment was conducted (> 4,000 measurements) that included personal and work-area submicrometer EC measurements covering the major job categories in the trucking industry. Exposure models based on terminal location in the United States were developed. Historical trends in ambient terminal EC were modeled based on historical trends in the coefficient of haze, a measurement of visibility interference in the atmosphere. In addition to changes in ambient exposure, the historical model accounted for changes in job-related exposures based on a comparison of EC measurement data obtained in 1988 through 1989 with the newly collected EC measurements. We used the risk estimates from analyses that excluded mechanics because in the study by Garshick et al. (2012) mechanics experienced significant historical changes in job duties that weakened the validity of extrapolation of the current exposure to historical estimates. In addition, the nature of exposure (intermittent exposure) was thought to be different from that of the other workers in study (i.e., longer periods of job-related exhaust exposure). However, we did include data from Garshick et al. (2012) that included mechanics in a sensitivity analysis of the obtained ERC (see Supplemental Material).

From the nested case–control miner study by Silverman et al. (2012), we used ORs for cumulative EC with a 15-year lag; we chose to use risk estimates from the nested case–control study instead of estimates from the cohort analysis (Attfield et al. 2012) because of their control for confounding, particularly from smoking, in the nested case–control study. The case–control study was nested within a cohort of 12,315 workers in eight non-metal mining facilities and included 198 lung cancer deaths and 562 incidence density–sampled controls. Respirable EC was estimated for each surface and underground job from the year of introduction of diesel-powered equipment in the facilities to 31 December 1997. Between 1998 and 2001, a detailed exposure assessment was conducted measuring personal respirable EC levels (> 700 measurements) covering the majority of job titles in the facilities (Stewart et al. 2010). These estimates were back-extrapolated for underground jobs per mine based on historical carbon monoxide measurement data and DEE-related determinants (e.g., diesel engine horsepower and ventilation rates).

*Meta-regression*. From the three studies, we extracted study-specific categorical RR estimates for lung cancer mortality in association with different cumulative DEE exposure levels relative to the lowest category of exposure for each study (see Supplemental Material, Table S1). We used the midpoint of the range of each exposure category as a specific estimate of the cumulative exposure for each RR. For the highest exposure category, we calculated the midpoint as 5/3 times the lower bound of the category, as proposed by the U.S. Environmental Protection Agency in 2008 (Lenters et al. 2011). However, from the study by Silverman et al. (2012), we obtained the median cumulative exposure value for the upper category (Silverman DT, personal communication).

The meta-regression models applied consisted of a full linear model and a separate model that incorporated a natural spline function with prespecified knots at the 20th, 50th, and 80th percentiles.

The models can be described as

lnRR = β_0_ + β_1_(exposure) + σ*_u_*_0_^2^ + σ*_u_*_1_^2^ + σ*_e_*_0_^2^, [1]

where β_0_ is the common intercept across studies, β_1_ is the common linear slope or spline function associated with DEE exposure across studies, σ*_u_*_0_^2^ is the estimated variance of the intercept between studies, σ*_u_*_1_^2^ is the estimated variance of the slope between studies and σ*_e_*_0_^2^ is the variance of the individual risk estimates. For the spline models, an additional spline variable was estimated by using third-order polynomials to fit a nonlinear slope (Harrell 2001).

In the meta-regression models, the natural logarithm (ln) of each study RR was inversely weighted by its variance, and correlations among the category-specific RRs from each individual study were accounted for by estimating their covariance (Greenland and Longnecker 1992). To account for potential between-study heterogeneity, the regression models allowed for random study-specific intercepts and exposure effects.

*Sensitivity analyses*. The meta-regression was repeated in a series of sensitivity analyses that used alternative data from one of the three studies while keeping the information from the other two studies unchanged from the main analysis, as described in Supplemental Material, Table S2. From the study by Garshick et al. (2012), we used HRs from unlagged analyses and from analyses using a 10-year lag (vs. 5 years for the main analysis) and performed a third sensitivity analysis using HRs based on analyses that included mechanics (5-year lag). From the study by Silverman et al. (2012), we used ORs based on unlagged data (vs. a 15-year lag for the main analysis) and performed a second sensitivity analysis with the OR for the highest quartile of exposure (15-year lag) excluded. From the study by Steenland et al. (1998), we performed one sensitivity analysis based on ORs for unlagged exposures (vs. a 5-year lag).

In addition, we performed two sensitivity analyses that included estimates from the study by Mohner et al. (2013), including one using HRs from the original cohort analysis, and a second using ORs that were corrected for the high level of DEE in the referent exposure group (624 μg/m^3^ EC). This correction was made under the assumption that the OR for the Mohner et al. (2013) referent category could be adjusted upward based on the RR predicted for an average exposure of 624 μg/m^3^ according to the main meta-analysis (specifically, to OR = 2.0) and that this adjusted reference OR could be used to recalibrate the nonreference effect estimates and standard errors.

*Excess lifetime risk calculations*. The excess lifetime risk (ELR) of lung cancer mortality associated with exposure to DEE was estimated using life table techniques accounting for all-cause mortality, applying an adaptation of the method described in a report by the Committee on the Biological Effects of Ionizing Radiation (National Research Council 1988). ELR was calculated through 80 years of age according to several different exposure scenarios. For occupational exposure, we assumed an exposure from 20 to 65 years of age, as typically done in occupational risk assessment, with average EC exposures of 25, 10, and 1 μg/m^3^. In addition, we estimated the ELR for environmental exposure from birth to 80 years of age to an average EC exposure of 0.8 μg/m^3^. All exposures were lagged 5 years. Average occupational EC exposures of 25 μg/m^3^ have been described for diesel mechanics, 10 μg/m^3^ for construction workers, and 1 μg/m^3^ for professional drivers (Pronk et al. 2009). Median ambient air EC levels of 0.8 μg/m^3^ (1.02 ×10^–5^/m black carbon) have been reported for metropolitan areas (Gan et al. 2013)

Background all-cause mortality (both sexes combined) were obtained from 2009 U.S. vital statistics [Centers for Disease Control and Prevention (CDC) 2014] and used to estimate the probability of surviving each 5-year age interval. In addition, we obtained lung cancer mortality rates for 2009 (CDC 2014) that were stratified by 5-year age groups and used to estimate the cumulative probability of dying from lung cancer in each 5-year age interval, conditional on not dying from other causes. These age-specific probabilities of lung cancer mortality were then summed across age groups to estimate the background lifetime (up to age 80 years) risk of dying from lung cancer in the absence of exposure to DEE. Next we estimated age-specific probabilities of lung cancer mortality in populations with occupational or environmental DEE exposure by multiplying each age-specific background lung cancer mortality rate by the RR from our primary exposure–response meta-analysis for the cumulative occupational or environmental DEE exposure level estimated for that age group. We estimated cumulative exposures for each age group assuming a constant exposure intensity (at the level assumed for the exposure scenario being evaluated) that accumulated daily, with a 5-year lag (e.g., exposure started at 25 years of age for occupational exposure and at age 5 years for environmental exposure). We chose a 5-year lag for our ELR analysis because a 5-year lag was reported to provide the best fitting model by two of the three studies (Garshick et al. 2012; Silverman et al. 2012; Steenland et al. 1998). As for the unexposed population, we summed the age-specific probabilities of lung cancer mortality to estimate the lifetime (up to 80 years of age) risk of dying from lung cancer among those exposed to DEE. Finally, we derived the ELR as

ELR = (risk_unexposed_ – risk_exposed_) ÷ (1 – risk_unexposed_), [2]

where risk_exposed_ and risk_unexposed_ represent the estimated lifetime risks of lung cancer mortality among those with and without DEE exposure, respectively. In addition to estimating ELRs for occupational exposures from 20 to 65 years of age, consistent with assumptions commonly used for regulatory purposes, we also derived ELRs for shorter occupational exposure scenarios (10 and 20 years with start of exposure at 20 years of age).

*Estimated proportion of lung cancer deaths attributable to DEE*. We used the RRs derived from the meta-regression at 70 years of age, to estimate the attributable fraction (AF) of lung cancers due to ever-exposure to DEE either in the environmental or occupational setting in the two countries (the United States and the United Kingdom) where we had data on the proportion of the population ever-exposed to DEE occupationally.

We estimated the AF of lung cancer mortality due to environmental exposure at 70 years of age, the approximate median age of lung cancer mortality in the United States in 2006–2010 (National Cancer Institute 2014). Information on environmental exposures is limited, but we assumed an average ambient EC concentration of 0.8 μg/m^3^ as estimated for 1994–1998 by Gan et al. (2013) for metropolitan Vancouver, British Columbia, Canada. An average exposure of 0.8 μg/m^3^ would result in a cumulative exposure at 70 years of age of 54-μg/m^3^-years, after accounting for a 5-year lag. Based on the meta-risk function, we can predict an RR of 1.05 for the exposed population. We then estimated the AF as follows:

AF = (risk_exposed_ – risk_unexposed_) ÷ risk_exposed_, [3]

which is equivalent to

AF = (RR – 1) / RR [4]

(Steenland and Armstrong 2006).

To estimate the AF of lung cancer mortality due to occupational exposures at 70 years of age, we assumed that approximately 5% (12 million of 230 million) of the adult U.S. population has been occupationally exposed to DEE based on data for the United States (Driscoll et al. 2005) that has recently been updated (Driscoll T, personal communication). Similarly, we assumed that 5% of the adult U.K. population is or has been occupationally exposed to DEE based on an estimate derived by other investigators using similar methodology (Brown et al. 2012).

Cherrie et al. (2011) estimated that 80% of the DEE exposed workers in the European Union can be regarded as low-exposed workers, whereas 20% would be regarded as high exposed (e.g., workers in mining, construction, and diesel mechanics). Based on the work of Pronk et al. (2009), Cherrie et al. (2011) estimated that the EC exposure concentrations in this high-exposed group would be on average 13 μg/m^3^. Assuming an overall log-normal distribution with a geometric SD of 3.0, we estimated the EC exposure for the low-exposed group to be 3 μg/m^3^ (Kromhout et al. 1993). Average occupational exposures of 3 μg/m^3^ and 13 μg/m^3^ from 20 to 65 years of age would result in cumulative exposures of 135- and 585-μg/m^3^-years at 70 years of age (using a 5-year lag). As for environmental exposures, to derive RRs for each exposure group, we multiplied the cumulative exposure (54-μg/m^3^-years by 70 years of age) by the slope factor from our meta-regression analysis for a 1-μg/m^3^ increase in cumulative exposure. We estimated the AF for occupational exposures at multiple levels of exposure as

AF = Σ*p_i_*(RR*_i_* – 1)/[Σ*p_i_*(RR*_i_* – 1) + 1] [5]

(Steenland and Armstrong 2006), where *p* represents the proportion of the general adult population with cumulative exposure to DEE at level *i*, and RR*_i_* represents the RR associated with cumulative exposure at level *i* (i.e., the meta-analysis RR × *i*).

## Results

**Figure 1:**
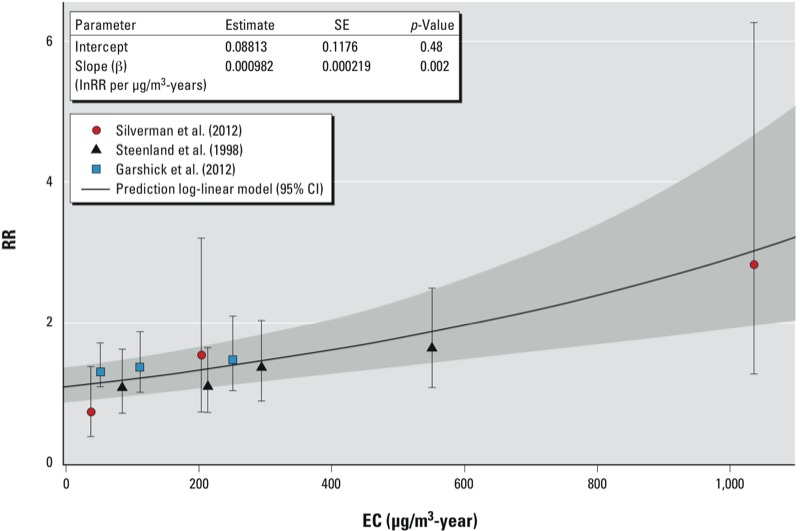
Predicted exposure–response curve based on a log-linear regression model using RR estimates from three cohort studies of DEE and lung cancer mortality. Individual RR estimates [based on HRs reported by Garshick et al. (2012) or ORs reported by Silverman et al. (2012) and Steenland et al. (1998)] are plotted with their 95% CI bounds indicated by the whiskers. The shaded area indicates the 95% CI estimated based on the log-linear model. The insert presents the estimates of the intercept and beta slope factor, the SE of these estimates, and the associated *p*-values.

The 10 extracted risk estimates from the three cohorts studied covered a cumulative exposure range, based on midpoints of the categories, from 37- to 1,036-μg/m^3^-years (see Supplemental Material, Table S1). The linear model (Figure 1) and the spline meta-regression model (data not shown) fit the data well, with virtually equivalent curves. Therefore, we present only the linear curve here, as it is a more parsimonious model with a lower Akaike information criterion (9.9 vs. 22.4, respectively). Slope factors (i.e., the lnRR estimated for a 1-μg/m^3^-year increase in EC) for the three studies included in the meta-analysis were within a factor of two, and 95% confidence intervals (CIs) largely overlapped (Table 1). The combined slope estimate was 0.00098 (95% CI: 0.00055, 0.00141).

**Table 1:** Exposure–response estimates (lnRR for a 1-μg/m^3^ increase in EC) from individual studies and the primary combined estimate based on a log-linear model.

Model^*a*^	Intercept	β (95%CI)
All studies combined	0.088	0.00098 (0.00055, 0.00141)
Silverman et al. (2012) only	–0.18	0.0012 (0.00053, 0.00187)
Steenland et al. (1998) only	–0.032	0.00096 (0.00033, 0.00159)
Garshick et al. (2012) only	0.24	0.00061 (–0.00088, 0.00210)
^***a***^Log-linear risk model (lnRR = intercept + β × exposure). Exposure defined as EC in μg/m^3^-years.		

Combined slope estimates based on the sensitivity analyses were generally consistent with the primary estimate, ranging from 0.00061 (95% CI: 0.00019, 0.00103) when data from the study by Silverman et al. (2012) of miners were unlagged, to 0.0011 (95% CI: 0.00040, 0.00172) when the RR for the highest quartile of exposure in Silverman et al. (2012) was excluded (see Supplemental Material, Table S3 and Figure S1). Combined estimates also were similar when data from the study by Mohner et al. (2013) were included in the meta-analysis.

For occupational exposures of 25-, 10-, and 1-μg/m^3^ EC over 45 years, assuming a 5-year lag, we estimated excess lifetime lung cancer mortality of 689, 200, and 17 deaths per 10,000 individuals (Table 2). For environmental exposures, assuming an average exposure of 0.8 μg/m^3^ over 80 years (with a 5-year lag), we estimated 21 excess lung cancer deaths per 10,000 individuals. Corresponding estimates for occupational exposures over 20 years were 252, 87, and 8 deaths per 10,000, and for occupational exposures over 10 years were 112, 41, and 4 deaths per 10,000.

**Table 2:** Excess lifetime risk per 10,000 for several exposure levels and settings, United States in 2009.

Exposure setting	Average EC exposure (μg/m^3^)	Excess lifetime risk through age 80 years (per 10,000)
Worker exposed, age 20–65 years	25	689
Worker exposed, age 20–65 years	10	200
Worker exposed, age 20–65 years	1	17
General public, age 5–80 years	0.8	21
Based on linear risk function, lnRR = 0.00098 × exposure, assuming a 5-year lag, using age-specific (5-year categories) all cause and lung cancer mortality rates from the United States in 2009 as referent.		

For average occupational exposures of 3 μg/m^3^ and 13 μg/m^3^ (Kromhout et al. 2000), the corresponding RRs at 70 years of age from our regression results are 1.14 and 1.78, respectively. The RR for an average environmental exposure of 0.8 μg/m^3^ at 70 years of age is 1.05. Combining these RRs with the estimated proportions of the population exposed, we estimated AFs of lung cancer deaths at 70 years of age due to environmental and occupational DEE exposures in the United States and the United Kingdom to be 4.8% and 1.3%, respectively. Combining the AFs for environmental and occupational exposures results in an overall AF of approximately 6% in the United States and the United Kingdom, which translates to about 9,000 annual lung cancer deaths in the United States and 2,000 annual lung cancer deaths in the United Kingdom that may be attributable to DEE.

## Discussion

Diesel engines were initially used predominantly to power heavy-duty equipment, with trains converting to diesel locomotives mainly after World War II (Laden et al. 2006) and with heavy-duty trucks converting to diesel primarily during the mid- to late 1950s (Davis et al. 2011). Dieselization of equipment in underground mines occurred mostly in the 1960s–1970s (Stewart et al. 2010). Diesel engines also are used in automobiles, especially in Europe. Large groups in the general population living in urban areas or close to highways are exposed to DEE, albeit to lower levels than in most occupational settings (Gan et al. 2013; Pronk et al. 2009). Given that DEE is classified as a known human carcinogen (Benbrahim-Tallaa et al. 2012), the impact of both occupational and environmental exposures on the overall lung cancer burden is potentially significant.

Currently EC is regarded as the best available proxy measure of DEE exposure in occupational settings (Birch and Cary 1996). We identified four studies that expressed the risk of lung cancer mortality by cumulative EC exposure. Of these studies, we retained three studies in the meta-regression and excluded one study because of methodological shortcomings. The retained studies were a study of non-metal miners (Silverman et al. 2012) and two independent studies of trucking industry workers (Garshick et al. 2012; Steenland et al. 1998).

Our estimates of the three study-specific slope factors (i.e., the lnRR for a 1-μg/m^3^-year increase in EC) ranged from 0.00061 (95% CI: 0.00019, 0.00102) to 0.0012 (95% CI: 0.00053, 0.00187), and CIs largely overlapped among the individual estimates. Furthermore, results of sensitivity analyses based on alternative results (e.g., using different exposure lags) from the individual studies, and inclusion of data from a study of potash miners (Mohner et al. 2013), which ranged from lnRR 0.00061 to 0.0011 for a 1-μg/m^3^-year increase in EC, were not substantially different from our main estimate of 0.00098 (95% CI: 0.00055, 0.00141). Hence, our estimated slope factor appeared to be relatively robust.

Interestingly, our slope estimate is roughly consistent with the risk of lung cancer mortality related to long-term population-based exposure to EC previously estimated by Janssen et al. (2011) based on a conversion of black smoke to EC for two European studies. Specifically, compared with no DEE exposure, the RR for a lifetime exposure at an average of 0.8 μg/m^3^ based on Janssen et al. (2011) would be approximately 1.03, compared with RR = 1.05 [exp(0.000982 × 70 years × 0.8 μg/m^3^)] based on our slope estimate (75 years exposure, 5-year lag).

We estimated excess lung cancer deaths per 10,000 individuals for lifetime environmental exposure and for average lifetime occupational exposure levels between 1 and 25 μg/m^3^. Estimated numbers of excess lung cancer deaths for occupational exposures of 45 years ranged from 17 to 689 per 10,000. These ELRs exceed the U.S. Occupational Safety and Health Administration and the European Union Scientific Committee on Occupational Exposure Limits typical goal of limiting ELR of disease for exposed workers to below 1/1,000 based on a lifetime exposure at an average exposure level. Workers in the trucking, railroad, and mining industries have been and still are often exposed to EC levels in these exposure ranges (Coble et al. 2010; Davis et al. 2011; Pronk et al. 2009; Vermeulen et al. 2010). With millions of workers currently exposed to such levels, and likely higher levels in the past, the impact on the current and future lung cancer burden could be substantial.

We estimated that environmental exposure in the general population (average EC, 0.8 μg/m^3^) resulted in an estimated excess lifetime risk of 21 additional lung cancer deaths per 10,000 individuals as compared to an unexposed population. With the high prevalence of such levels of exposure in the general population of urban areas, the contribution to the lung cancer burden could be substantial.

We believe that it is appropriate to use U.S. lung cancer rates, unadjusted for smoking, in the ELR calculations under the assumption that smoking does not modify the association between DEE and lung cancer mortality. Different smoking habits in the general population (from which we derived our lung cancer mortality rates), compared with the cohorts (from which we derived our exposure–response function) would not affect our estimates of excess lifetime mortality if the assumption of no effect modification by smoking were correct. If smoking does modify the effect of DEE, data from one study (Silverman et al. 2012) suggest that at high DEE exposure, nonsmokers may have a higher RR per unit of exposure than smokers, which implies that our ELR would be an underestimate, since historically blue-collar worker populations are known to have lower percentages of nonsmokers than the general population (Nelson et al. 1994).

We estimated that approximately 1.3% and 4.8% of annual lung cancer deaths at 70 years of age in the United States and the United Kingdom are due to past occupational and environmental DEE exposures, respectively. These estimates are far from precise and depend on broad assumptions about proportions exposed to different levels of DEE and the duration of occupational exposures. However, our AF estimate for occupational DEE exposure is consistent with an AF of 1.5% estimated by Brown et al. (2012) for the United Kingdom. In addition, our AF estimate for environmental DEE exposure is generally consistent with previous estimates for traffic-related air pollution and lung cancer mortality and incidence (5–7%) (Cohen et al. 2005; Vineis et al. 2007).

There are several points about our meta-regression worth noting. First, the study data on which our meta-regression was based are limited, resulting in inherent uncertainty in the obtained slope estimates. Formal tests of heterogeneity of estimates among the studies were of limited value due to the small number of data points for each study. Second, we extrapolated our results, which, based on spline models (data not shown), were largely linear on the log RR scale, to exposures which in some cases are lower than exposures observed in our occupational studies. However the extrapolation is not large, because exposures as low as 1 μg/m^3^ are present in our occupational data. Third, we recognize that not all EC in the general environment is from DEE, and as such the EC exposures in the occupational and general environment could be qualitatively different. Fourth, our estimates of the AF are based on broad assumptions regarding exposure distributions in occupational and environmental settings. However, available data to support these assumptions are limited. Fifth, estimates from the studies used in our meta-analysis differed with regard to the exposure lag time, with two studies using a 5-year lag and the third a 15-year lag. However, the combined slopes based on sensitivity analyses were generally consistent with our primary estimate when we used unlagged estimates from each study or estimates derived using a 10-year lag from one of the studies. Sixth, there is considerable uncertainty inherent in retrospective exposure assessment. Nonetheless, in all three of our key studies, considerable resources were devoted to this task, and a relative large number of air samples were available in each study. Seventh, we were not able to investigate other model forms in our meta-regression, beyond the linear and spline curves because of the limited number of data points. If nonlinear exposure–response curves were actually a better fit (e.g., attenuation at higher exposures, for which there is some evidence in Silverman et al. (2012), then this might change the estimate burden of disease due to DEE.

Our estimates suggest that stringent occupational and environmental standards for DEE should be set. Fortunately, increasingly stringent on-road emission standards for diesel engines have been introduced in the United States and the European Union (U.S. 2010 and Euro 6 standards), with other countries (e.g., China, India, Brazil) following with a delay of about 5–10 years (Scheepers and Vermeulen 2012). These regulations have resulted in the recent introduction of new diesel engine technologies (integration of the wall-flow diesel particulate filter and diesel oxidation catalyst) that on a per-kilometer basis achieve a > 95% reduction of particulate mass and nitrogen oxides emissions (Scheepers and Vermeulen 2012). However, emission standards for off-road vehicles and industrial applications are generally introduced after those for on-road vehicles and, therefore, many off-road applications were still largely uncontrolled in 2000. It should also be noted that although new diesel engines are available, it will take still many years before they have a significant penetration into the diesel engine fleet, especially in less developed countries (Scheepers and Vermeulen 2012).

## Conclusion

In a recent *IARC Monograph* evaluation, DEE was classified as a known human lung carcinogen (Benbrahim-Tallaa et al. 2012). Based on a meta-regression derived from three occupational studies critical to the IARC evaluation (Benbrahim-Tallaa et al. 2012), we estimated substantial excess lifetime lung cancer risks for several occupational and environmental exposure scenarios. Each are above the usual occupational and environmental limits used in Europe and the United States, which are set at 1/1,000 and 1/100,000 based on lifetime exposure for the occupational and general population, respectively.

## Supplemental Material

(639 KB) PDF
