# Supplementary material for: Exposure-Response Estimates for Diesel Engine Exhaust and Lung Cancer Mortality Based on Data from Three Occupational Cohorts
Source: Environ Health Perspect. 2013 Nov 22;122(2):172–7. doi: 10.1289/ehp.1306880 (PMC3915263; doi:10.1289/ehp.1306880)
Supplement: (639 KB) PDF [file ehp.1306880.s001.508.pdf]

Environmental Health Perspectives doi:10.1289/ehp.1306880

Supplemental Material

Exposure-Response Estimates for Diesel Engine Exhaust and Lung Cancer Mortality Based on Data from Three Occupational Cohorts

Roel Vermeulen, Debra T. Silverman, Eric Garshick, Jelle Vlaanderen, Lützen Portengen, and Kyle Steenland

Table of Contents

Supplemental Material, Table S1……………………………………………..page 2

Sensitivity Analyses of the Meta Exposure Response Curve…………………...page 4

Supplemental Material, Table S2……………………………………………..page 5

Supplemental Material, Table S3……………………………………………..page 6

Supplemental Material, Figure S1……………………………………………page 7

References…………………………...…………………………………………...page 8

Table S1. Extracted relative risk estimates from four studies with diesel exhaust exposure estimates expressed by cumulative elemental carbon (EC) used in the primary meta-regression and subsequent sensitivity analysis.

Study	Reported EC exposure category (µg/m³-years)	Assigned exposure (µg/m³-years)	Lag time (years)	Relative risk (95% CI)	Analysis	Comments
Garshick et al. 2012						
	0 - < 30.9	15.5	5	1 (1, 1)	Primary	Reference category; excluding mechanics
	30.9-71.7	51.3	5	1.31 (1.1, 1.71)	Primary	Excluding mechanics
	71.7-150.3	111	5	1.38 (1.02, 1.87)	Primary	Excluding mechanics
	≥ 150.3	250.5	5	1.48 (1.05, 2.1)	Primary	Excluding mechanics
	0-44.2	22.1	0	1 (1, 1)	Sensitivity	Reference category; excluding mechanics
	44.2-88.4	66.3	0	1.25 (0.99, 1.6)	Sensitivity	Excluding mechanics
	88.4-173	132.6	0	1.3 (0.99, 1.72)	Sensitivity	Excluding mechanics
	> 173	288.3	0	1.24 (0.89, 1.71)	Sensitivity	Excluding mechanics
	0 - <13.9	7.0	10	1 (1, 1)	Sensitivity	Reference category; excluding mechanics
	13.9-49.7	31.8	10	1.17 (0.88, 1.57)	Sensitivity	Excluding mechanics
	49.7-119.7	84.7	10	1.26 (0.9, 1.78)	Sensitivity	Excluding mechanics
	≥ 119.7	199.5	10	1.41 (0.95, 2.11)	Sensitivity	Excluding mechanics
	0 - < 30.9	15.5	5	1 (1, 1)	Sensitivity	Reference category; including mechanics
	30.9-71.7	51.3	5	1.3 (1.01, 1.68)	Sensitivity	Including mechanics
	71.7-150.3	111	5	1.35 (1.01, 1.81)	Sensitivity	Including mechanics
	≥ 150.3	250.5	5	1.36 (0.98, 1.89)	Sensitivity	Including mechanics
Silverman et al. (2012)						
	0 - < 3	1.5	15	1 (1, 1)	Primary	Reference category
	3 - < 72	37.5	15	0.74 (0.4, 1.38)	Primary	
	72 - < 536	204	15	1.54 (0.74, 3.2)	Primary	
	≥ 536[a]	1036	15	2.83 (1.28, 6.26)	Primary	

Study	Reported EC exposure category (μg/m^3-years)	Assigned exposure (μg/m^3-years)	Lag time (years)	Relative risk (95% CI)	Analysis	Comments
	0-19	9.5	0	1 (1, 1)	Sensitivity	Reference category
	19-246	132.5	0	0.87 (0.48, 1.59)	Sensitivity	
	246-964	605	0	1.5 (0.67, 3.36)	Sensitivity	
	> 964	1606	0	1.75 (0.77, 3.97)	Sensitivity	
Steenland et al. (1998)						
	0	0	5	1 (1, 1)		Reference category
	0-169	84.5	5	1.08 (0.72, 1.63)	Primary	
	169-257	213	5	1.1 (0.74, 1.65)	Primary	
	257-331	294	5	1.36 (0.9, 2.04)	Primary	
	≥ 331	551.7	5	1.64 (1.09, 2.49)	Primary	
	0	0	0	1 (1, 1)	Sensitivity	Reference category
	0-174	87	0	1.2 (0.79, 1.81)	Sensitivity	
	174-268	221	0	1.16 (0.77, 1.75)	Sensitivity	
	268-360	314	0	1.39 (0.91, 2.11)	Sensitivity	
	> 360	600	0	1.72 (1.11, 2.64)	Sensitivity	
Mohner et al. (2013)						
	< 983	624	5	1 (1, 1)	Sensitivity	Reference category; reported RRs
	983-1550	1279	5	1.77 (0.85, 3.69)	Sensitivity	Reported RRs
	> 1550	2375	5	1.04 (0.47, 2.27)	Sensitivity	Reported RRs
	< 983	624	5	1 (1, 1)	Sensitivity	Reference category; adjusted RRs
	983-1550	1279	5	3.57 (1.71, 7.44)	Sensitivity	Adjusted RRs
	> 1550	2375	5	2.1 (0.96, 4.58)	Sensitivity	Adjusted RRs

[a]Highest exposure category was omitted in one of the sensitivity analyses (see Supplemental Material, Table S2).

Sensitivity Analyses of the Meta-Exposure-Response Curve

The meta-regression was repeated allowing for different risk sets per individual study (see Supplemental Material, Table S2). For the Garshick et al. study (Garshick et al. 2012) we allowed for sensitivity analyses including alternative lag times of 0 and 10 years (primary 5 years), and for risk estimates obtained with inclusion of mechanics (sensitivity analyses #2, 3, and 4, respectively). For the Silverman et al. study (Silverman et al. 2012) we allowed for alternative lag times (0 instead of 15 years) and excluded the highest point of the study (sensitivity analyses #5 and 6, respectively). For the Steenland et al. study (Steenland et al. 1998) the primary ERC was based on a lag time of 5 years. ERC was re-estimated based on the risk estimates obtained with a lag-time of 0 years (sensitivity analysis #7). In addition, we re-estimated the primary ERC with inclusion of the Mohner et al. study (Mohner et al. 2013) including the original risk estimates from that study, and the bias-corrected risk estimates accounting for the high DEE exposure of the referent group (624 $\mu g/m^3$ EC) based on our meta-regression results without Mohner et al. included (sensitivity analyses 8 and 9, respectively). Results of the model parameter estimates are presented in Supplemental Material, Table S3 and graphically in Supplemental Material, Figure S1.

Table S2. Overview of sensitivity analyses conducted.

Sensitivity analysis	Garshick et al. (2012)	Silverman et al. (2012)	Steenland et al. (1998)	Mohner et al. (2013)
1 (primary analyses)	5-yr lag; excl mechanics	15-yr lag	5-yr lag	Not included
2	0-yr lag; excl mechanics[a]	15-yr lag	5-yr lag	Not included
3	10-yr lag; excl mechanics[a]	15-yr lag	5-yr lag	Not included
4	5-yr lag; incl mechanics[a]	15-yr lag	5-yr lag	Not included
5	5-yr lag; excl mechanics	0-yr lag[a]	5-yr lag	Not included
6[b]	5-yr lag; excl mechanics	15-yr lag[a]	5-yr lag	Not included
7	5-yr lag; excl mechanics	15-yr lag	0-yr lag[a]	Not included
8	5-yr lag; excl mechanics	15-yr lag	5-yr lag	Original RRs included[a]
9	5-yr lag; excl mechanics	15-yr lag	5-yr lag	Adjusted RRs included[a]

[a]Shaded cells indicate the alternative as compared to the primary analysis (model 1). [b]Analysis excluding the relative risk estimate for the highest exposure category (>≥ 536 μg/m^3-years) from Silverman et al. (2012).

Table S3. Parameter estimates (point estimate, standard error and p-value) of alternative models as conducted as part of the sensitivity analyses.

	Intercept			Slope		
**Sensitivity analysis**	**Estimate**	**95%CI**	**Pr> |t|**	**Estimate**	**(95%CI)**	**Pr> |t|**
1 (primary model)	0.08813	-0.1423, 0.3186	0.475	0.000982	0.00055, 0.0014	0.0021
2	0.06732	-0.1286, 0.2633	0.5198	0.000909	0.00049, 0.0013	0.003
3	0.0399	-0.1755, 0.2553	0.7259	0.001021	0.00059, 0.0015	0.0018
4	0.09719	-0.1094, 0.3038	0.3835	0.000936	0.00051, 0.0014	0.0026
5	0.1326	-0.0879, 0.3531	0.2726	0.000608	0.00019, 0.0010	0.0211
6[a]	0.07652	-0.1647, 0.3178	0.554	0.00106	0.00040, 0.0017	0.0159
7	0.08352	-0.1209, 0.2879	0.4465	0.000927	0.00042, 0.0014	0.0074
8	0.121	-0.1024, 0.3444	0.3133	0.000646	0.00000, 0.0012	0.0559
9	0.1371	-0.0677, 0.3419	0.2187	0.000713	0.00030, 0.0011	0.0068

[a]Analysis excluding highest risk estimate from Silverman et al. 2012.

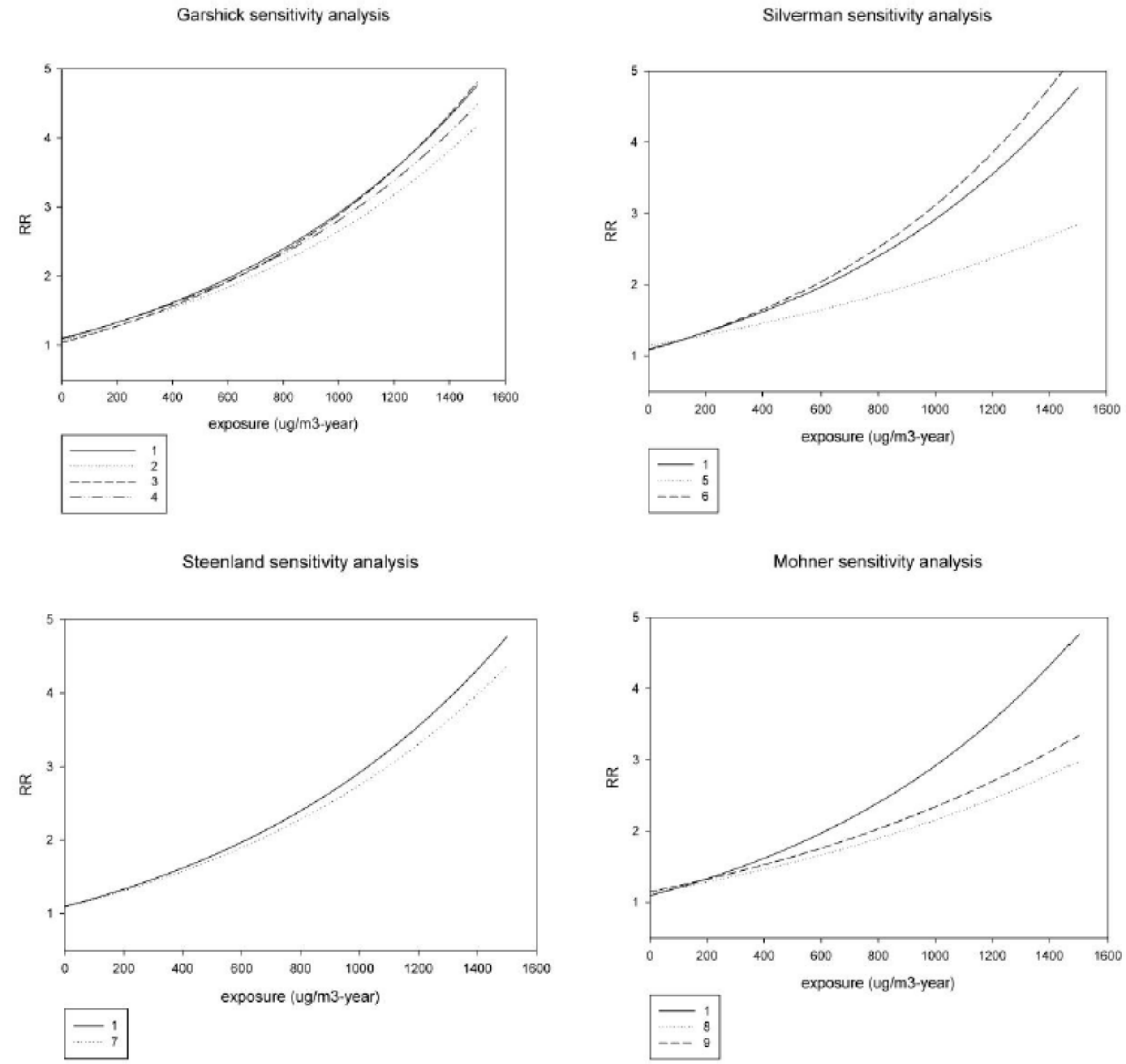

Figure S1. Predicted meta-exposure response curve based on a log-linear regression model using risk estimates from the sensitivity analyses as described in Supplemental Material, Table S2 and Table S3**.**

References

Garshick E, Laden F, Hart JE, Davis ME, Eisen EA, Smith TJ. 2012. Lung cancer and elemental carbon exposure in trucking industry workers. Environ Health Perspect 120:1301-1306.

Mohner M, Kersten N, Gellissen J. 2013. Diesel motor exhaust and lung cancer mortality: reanalysis of a cohort study in potash miners. Eur J Epidemiol 28:159-168.

Silverman DT, Samanic CM, Lubin JH, Blair AE, Stewart PA, Vermeulen R et al. 2012. The Diesel Exhaust in Miners Study: A Nested Case-Control Study of Lung Cancer and Diesel Exhaust. J Natl Cancer Inst

Steenland K, Deddens J, Stayner L. 1998. Diesel exhaust and lung cancer in the trucking industry: exposure-response analyses and risk assessment. Am J Ind Med 34:220-228.
